# Evolution of Mitochondrial Power in Vertebrate Metazoans

**DOI:** 10.1371/journal.pone.0098188

**Published:** 2014-06-09

**Authors:** Yasuhiro Kitazoe, Masashi Tanaka

**Affiliations:** 1 Center of Medical Information Science, Kochi Medical School, Nankoku, Kochi, Japan; 2 Department of Genomics for Longevity and Health, Tokyo Metropolitan Institute of Gerontology, Tokyo, Japan; University of Texas Health Science Center at San Antonio, United States of America

## Abstract

**Background:**

Basal metabolic rate (*BMR*) has a very strong body-mass (*M*) dependence in an individual animal group, and *BMR* per unit mass (*msBMR*) converges on a markedly narrow range even across major taxonomic groups. However, it is here a basic question in metazoan biology how much *BMR* per unit mitochondrion (*mtBMR*) changes, and then whether *mtBMR* can be related to the original molecular mechanism of action of mt-encoded membrane proteins (MMPs) playing a central role in cellular energy production.

**Methodology/Principal Findings:**

Analyzing variations of amino-acid compositions of MMPs across 13 metazoan animal groups, incorporating 2022 sequences, we found a strong inverse correlation between Ser/Thr composition (*STC*) and hydrophobicity (*HYD*). A majority of animal groups showed an evolutionary pathway of a gradual increase in *HYD* and decrease in *STC*, whereas only the deuterostome lineage revealed a rapid decrease in *HYD* and increase in *STC*. The strongest correlations appeared in 5 large subunits (ND4, ND5, ND2, CO1, and CO3) undergoing dynamic conformational changes for the proton-pumping function. The pathway of the majority groups is well understood as reflecting natural selection to reduce *mtBMR*, since simply raising *HYD* in MMPs (surrounded by the lipid bilayer) weakens their mobility and strengthens their stability. On the other hand, the marked decrease in *HYD* of the deuterostome elevates *mtBMR*, but is accompanied with their instability heightening a turnover rate of mitochondria and then cells. Interestingly, cooperative networks of interhelical hydrogen-bonds between motifs involving Ser and Thr residues can enhance MMP stability.

**Conclusion/Significance:**

This stability enhancement lowers turnover rates of mitochondria/cells and may prolong even longevity, and was indeed founded by strong positive correlations of *STC* with both *mtBMR* and longevity. The lowest *HYD* and highest *STC* in Aves and Mammals are congruent with their very high *mtBMR* and long longevity.

## Introduction

Because the basal metabolic rate (*BMR*) is a fundamental currency to sustain metazoan life, it must be profoundly relevant to the mt power in energy production. However, its strong mass (*M*)-dependence makes unclear the existence of a relationship between *BMR* and this mitochondrial (mt) energy power across major taxonomic groups. A recent allometric study reports that the mass specific BMT (*msBMR*) converges on a markedly narrow range in these groups [1]. This viewpoint of the normalized energy inclines us to convert *msBMR* into the mt *BMR* (*mtBMR*) per unit mitochondrion which stands for the mt energy power, since the conversion can be done when *msBMR* includes the falling effect of the mt density (the mean number of mitochondria per unit cell) with increasing *M* [2], [3]. It is intriguing to estimate how much *mtBMR* changes across taxonomic groups, because recent structural studies report a high degree of sequence conservation of the membrane integral central subunits [4], [5], the mechanism of which is therefore likely to be similar throughout species [6].

The first step to relate *mtBMR* to the mt energy production power is to investigate the molecular structure of mt-encoded membrane proteins (MMPs) by using a number of amino acid sequences which are available in the NCBI database [7] (the accession numbers of these sequences are listed up in Table S1). The great majority of MMPs belongs to the 3 proton-pumping complexes of I, III and IV. Recent structural studies suggest that proton translocation in complex I requires large dynamic conformational changes across several subunits [6], [8], [9]. Likewise, the two large subunits of complex IV, i.e., CO1 and CO3, transfer protons across the membrane via conformational changes induced by electron transport [10]–[12].

MMPs are mostly embedded in the hydrophobic environment of the lipid bilayer, and their amino acid composition is primarily hydrophobic, with approximately 90–95% of these amino acids being non-polar. Therefore, the degree (mobility) of their conformational changes much depends on hydrophobicity (*HYD*): Raising *HYD* weakens their mobility and strengthens their stability according to the trade-off relation between mobility and stability [13]. Interestingly, a recent study of membrane proteins reports that the dynamic conformational stability of membrane helices can be typically enhanced by cooperative networks of interhelical hydrogen bonds between moderately polar residues, notably Ser and Thr [13]–[15]. The above-mentioned two features of *HYD* and Ser/Thr composition (*STC*) allow us to conceive a basic scenario of the metazoan evolution that lowering *mtBMR* (on the basis of the multicellular effect) requires less dynamic conformational changes of MMPs which induce an increase in *HYD* and a decrease in *STC*.

Here we report that most members of major animal groups follow this evolutionary scenario. However, the deuterostome lineage reveals the converse, i.e., rapid increases in *STC* and *mtBMR*, and a rapid decrease in *HYD* toward the endpoints (Aves and Mammals) of this lineage. Aves and Mammals seem ready to power up the mt energy by activating dynamical conformational changes of MMPs and still then enhance stability (durability) of them by increasing helix-helix interactions. This durability lowers turnover rates of mitochondria and cells, and may prolong longevity of organisms. Indeed, a strong correlation between *STC* and maximum lifespan (*MLS*) (observed in a previous vertebrate analysis [16]) was found to extend as a global rule beyond vertebrates across metazoans.

## Materials and Methods

### Derivation of *mtBMR* from *msBMR*

The allometric scaling law provides a very strong correlation between *BMR* and *M* in each animal group, and is expressed as *BMR =  C*•*M^α^* with an allometric exponent α and constant *C*. Makarieva et al. [1] well described a variation of *BMR* data across different animal groups by using *BMR* per unit mass (*msBMR*), i.e., *msBMR =  C*•*M* ^−(1−*α*)^

They reported that *msBMR* data across dramatically different life forms converge on a markedly narrow range. This unit-mass representation of *msBMR* implicitly means that an organism is approximately regarded as a homogeneous matter of standard (representative) cells: the number of cells in unit mass and also that of mitochondria (the mt density) in unit cell are invariant, respectively, although, in practice, metabolically active cells, such as those of the liver, kidneys, muscles, and brain, have hundreds or thousands of mitochondria [17]. Therefore, *msBMR* is proportional to *BMR* per unit cell (we put this proportional constant equal to 1.0). Next, to get *BMR* per unit mitochondrion, we divide *msBMR* by the factor *M* ^−β^ which takes into account the decreasing effect of the mt density with increasing *M* [2], [3]. Then we have *mtBMR* =  (*C/D*)•*M* ^− (1−α−β)^, which means that *mtBMR* decreases with increasing *M* more slowly than does *msBMR*. In this paper, we express *mtBMR* as follows: *mtBMR*  = *C*•*M* ^− (1−*α*)/*F*^. Here, *F* is with a new parameter to adjust the allometric scaling effect of the *M*-dependence. For simplicity, we put the proportional constant *D* equal to 1.0, since the value of *F* = 1.0 corresponds to *msBMR*. In a previous mammalian analysis, the value of *F* = 3.0 was selected as providing the strongest correlation between *mtBMR* and *MLS* [16]. In the present analysis, we redefine *M* as the mean value of the individual body masses in each animal group, to examine a relationship between *mtBMR* and amino acid compositions of MMPs in major taxonomic groups.

### Data retrieval

To select a hydrophobic domain in MMPs, we applied the primary structure analysis (ExPASy Proteomics Server; http://www.expasy.ch/), using a standard model for the hydrophobic score (*HYDSC*) given by Cowan and Whittaker [18]. We calculated the moving average, *S*(n), of *HYDSC*(m; m takes n-1, n, and n+1) around the n-th amino acid site in a protein and obtained a smooth function *S*(n) of n by repeating this procedure. As a result, *HYD* was defined as the average value of *S*(n) with *S*(n) >0.0 in all or selected proteins of a given species. The obtained *HYD* is suitable for examining correlations with other quantities of present interest, such as amino acid compositions and lifespan. We predicted the helix domain of MMPs by using SOSUI and TMHMM servers [19], [20].

## Results

### A) *HYD*-*TC* correlation within respective MMPs

By including 13 metazoan animal groups with many amino acid sequences (more than 20) in the NCBI database [7], we analyzed 13 MMPs with a score *S*>0 for their hydrophobic domain (Materials and Methods). As a result, we selected 4 MMP variables (*HYD*, *STC*, *TC* and *CC*) of amino acid compositions as having significant correlations with one another, and found that *HYD*-*TC* provided an especially strong correlation. Here, TC and CC denote the Thr and Cys compositions, respectively. Table 1 shows a list of MMPs in the order of strong correlations. The 3 large subunits of ND4, ND5, and ND2 in complex I appeared as the first group with the largest R^2^-values (R^2^>0.86). Likewise, 2 large subunits of CO1 and CO3 in complex IV appeared as the second group (with R^2^>0.78). These subunits just correspond to the proteins which require dynamic conformational changes for proton translocation [6], [8], [9]. The *TC*-*CC* correlation was appreciable in only these 2 proton-pumping complexes (with R^2^>0.40) undergoing dynamic conformational changes in their helices.

**Table 1:** *HYD*-*TC* and *TC*-*CC* correlations (R^2^) within respective proteins.

	ND4	ND5	ND2	CO1	CO3	ND1	ND3*	ATP8*	CYTB	ND4L*	CO2*	ATP6*	ND6*
**HYD-TC**	**0.89**	**0.89**	**0.87**	**0.81**	**0.78**	**0.68**	**0.68**	**0.65**	**0.60**	**0.58**	**0.57**	**0.37**	**0.03**
**TC-CC**	**0.60**	**0.61**	**0.64**	**0.41**	**0.49**	**0.38**	**0.32**	**0.25**	**0.26**	**0.11**	**0.29**	**0.63**	**0.42**

The * symbol denotes 2 subunits with weak correlations (ATP6 and ND6) and 4 subunits with small numbers (3 or less in humans) of helices (ND3, ATP8, ND4L, and CO2). The analysis includes the following 13 metazoan animal groups: Porifera, Cnidaria, Mollusca, Crustacea, Hexapoda, Chelicerata, Nematoda, Platyhelminthes, Echinodermata, Fishes, Amphibia, Eutheria, and Aves.

### B) Correlations between the MMP variables (*HYD*, *STC*, *TC* and *CC*)

**Figure 1:**
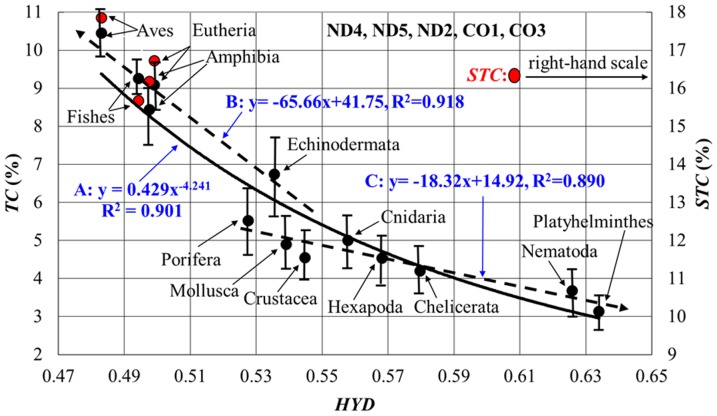
Global relationship between *TC* and *HYD* in MMPs of metazoan animals. Solid circles represent the average values of *HYD* and *TC* in each animal group, with the hydrophobic score S>0 (see Materials and Methods). The red circles show the *STC* values, which well describe the vertebrate lineage [16]. Such a strong correlation was also obtained by analyzing all 13 proteins (Figure S1). The correlation is totally well reproduced by a non-linear function (**A**: *TC* = 0.429•*HYD* ^−4.2045^ with R^2^ = 0.901), but it can be separately expressed by 2 regression lines with different slopes (**B**: the dotted line for the deuterostomes with R^2^ = 0.918) and (**C**: the dotted line for the other groups with R^2^ = 0.890). The error range of the x-axis (*HYD*) in an animal group can be estimated by moving the regression curve **A** in parallel along the y-axis so that the y-value of this curve may be equal to that of the solid circle of the group, since this error range of *HYD* may be roughly given by the x-axis values of the curve corresponding to the error range of the y-axis (*STC*).

**Figure 2:**
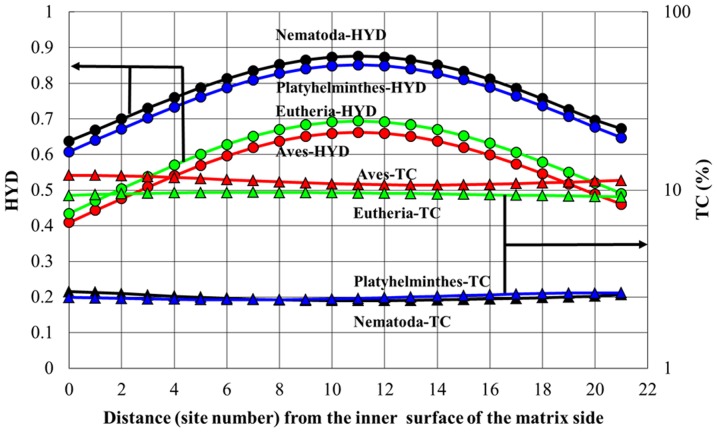
*HYD* and *TC* distributions in the mt inner membrane of ND2, ND4 and ND5. Four animal groups were selected as providing extreme situations of the hydrophobic distribution. This result was obtained by using SOSUI WWW server [19] and TMHMM Server [20] for the prediction of the secondary structure of proteins.

We investigated the intra-correlations between the MMP variables, by using the following 5 sets of proteins according to the order of strong correlations shown in Table 1: **1**) 3-protein set (ND4, ND5, ND2), **2**) 4-protein set (ND4, ND5, ND2, ND1), **3**) 5-protein set (ND4, ND5, ND2, CO1, CO3), **4**) 6-protein set (ND4, ND5, ND2, CO1, CO3, ND1), and **5**) 7-protein set (ND4, ND5, ND2, CO1, CO3, ND1, CYTB). Here, the 3-protein set included 39% of the total site number of the complete amino acid sequence in humans, and the 7-protein set, 76% of it. The 7-protein set did not include ND3 and ATP8 with small numbers of helices (3 or less in humans). As seen in Table 2, *TC* provided predominantly strong correlations with *HYD* in all protein sets, and the strongest correlation (R^2^ = 0.9) in the 5-protein set (Figure 1). In addition to this, *TC*-*CC*, *HYD*-*CC* and *HYD*-*STC* showed appreciable correlations. Here, we used the average values of *TC* and *HYD* in each animal group, in order to describe the correlation pattern lucidly (the raw data without the averaging procedure also showed a strong correlation of R^2^ = 0.9 (Figure S1). As a result, the *TC*-values in Aves and Eutheria with very high *BMR* were 2.5 fold larger than those of Nematoda and Platyhelminthes with very low *BMR*, and the *HYD* values of the former were decreased by about 22% compared with those of the latter. The validity of these estimations of *TC* and *HYD* was supported by speculating the *TC* and *HYD* distributions in the helix domain of the above-mentioned 4 animal groups (Figure 2).

**Table 2:** Correlations (R^2^) between the pairs of variables (*HYD*, *TC*, *STC*, *CC*, *TSN*, *mtBMR*, *msBMR* and *MLS*).

		3 proteins	4 proteins	5 proteins	6 proteins	7 proteins	mean
		<39%>	<47%>	<59%>	<66%>	<76%>	
***TC-HYD***	**N**	**0.8898**	**0.8939**	**0.8997**	**0.8958**	**0.8867**	**0.89318**
***TC-CC***	**N**	**0.6554**	**0.6916**	**0.6994**	**0.7264**	**0.6691**	**0.68838**
***HYD-CC***	**P**	**0.5295**	**0.6081**	**0.6367**	**0.5449**	**0.5337**	**0.57058**
***HYD-STC***	**N**	**0.5052**	**0.4803**	**0.2992**	**0.3503**	**0.3151**	**0.3901**
***STC-CC***	**N**	**0.2387**	**0.2298**	**0.209**	**0.1887**	**0.1551**	**0.20426**
***TSN-TC***	**P**	**0.9232**	**0.9014**	**0.8907**	**0.8019**	**0.6699**	**0.83742**
***TSN-HYD***	**N**	**0.7591**	**0.7364**	**0.7484**	**0.6491**	**0.7171**	**0.7221**
***STC-*ln*(mtBMR)***	**P**	**0.6434**	**0.6483**	**0.7048**	**0.6762**	**0.6751**	**0.66956**
***TC-*ln*(mtBMR)***	**P**	**0.4999**	**0.4995**	**0.4948**	**0.5159**	**0.5316**	**0.5083**
***HYD-*ln*(mtBMR)***	**N**	**0.3403**	**0.3206**	**0.3547**	**0.3379**	**0.3574**	**0.34218**
***CC-ln(mtBMR)***	**N**	**0.4041**	**0.3441**	**0.3401**	**0.3184**	**0.2851**	**0.33836**
***STC-*ln*(msBMR)***	**P**	**0.1937**	**0.1979**	**0.2804**	**0.2514**	**0.2428**	**0.23324**
***STC-*ln*(MLS)***	**P**	**0.7065**	**0.6861**	**0.5691**	**0.5691**	**0.5687**	**0.6944**
***CC-*ln*(MLS)***	**N**	**0.3732**	**0.3962**	**0.3447**	**0.3667**	**0.3441**	**0.3654**
***STC-*ln*(mtBMR·MLS)***	**P**	**0.8133**	**0.8052**	**0.7737**	**0.7561**	**0.7551**	**0.78068**

<n> denotes that *TSN* of each protein set occupies the n % of that of the complete amino acid sequence in Human. P and N stand for the positive and negative correlations, respectively. The best results in the respective correlation croups are denoted by italics.

### C) Correlations between MMP variables and total site number of amino acids

**Figure 3:**
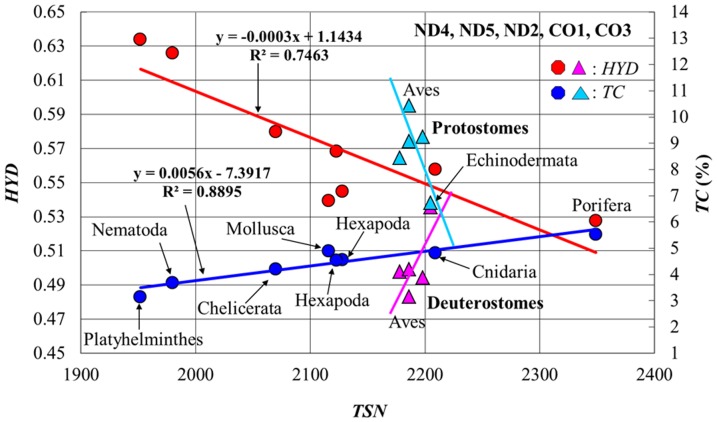
*HYD* and *TC* versus *TSN*. The regression lines for Deuterostomes and those for Protostomes were estimated separately. *HYD* and *TC* are expressed on the left and right ordinates, respectively.

We found that the total site number (*TSN*) of amino acids in a protein set steadily changes across the 13 animal groups (by 20% as a whole) and is a good index to describe the mutually contrasting evolutionary pathways of *TC* and *HYD* in metazoans (Figure 3). The *TSN* order of the animal groups (their relative *TSN*-dependence) was invariant in all protein sets (Figure S2). As a result, *TSN* strongly correlated with *TC* and *HYD*, when the deuterostomes were excluded (Table 2). Indeed, *TC* gradually decreased with decrease in *TSN* of many animal groups except for the deuterostomes (Figure 3, blue regression line), whereas *HYD* increased with a decrease in *TSN* (Figure 3, red regression line). On the other hand, *TC* and *HYD* in the deuterostome lineage presented rapidly increasing and decreasing trends with a decrease in *TSN* towards the terminal branch of Aves, clearly splitting from the 2 regression lines. This splitting pattern could be identified by looking at the *TC*-*HYD* relationship of Figure 1, because the non-linear regression curve **A** (*TC* = 0.429•*HYD* ^−4.241^ with R^2^ = 0.90 as a whole) was decomposed into a steep slope dotted-line **B** (*TC* = −65.66•*HYD*+41.75 with R^2^ = 0.92) for the deuterostomes and a slow slope dotted-line **C** (*TC* = −18.32•*HYD*+14.92 with R^2^ = 0.89) for the other animal groups. The splitting pattern of Figure 3 became compatible with a molecular (rRNA)-based phylogeny [21], in the point that the tree starts with the root of Porifera and splits into the two lineages of Deuterostomia and Protostomia via Cnidaria. In this way, the *TC*-*HYD* relationship globally reflected the evolutionary pathway of metazoans.

### D) Correlations between MMP variables and *mtBMR*

**Figure 4:**
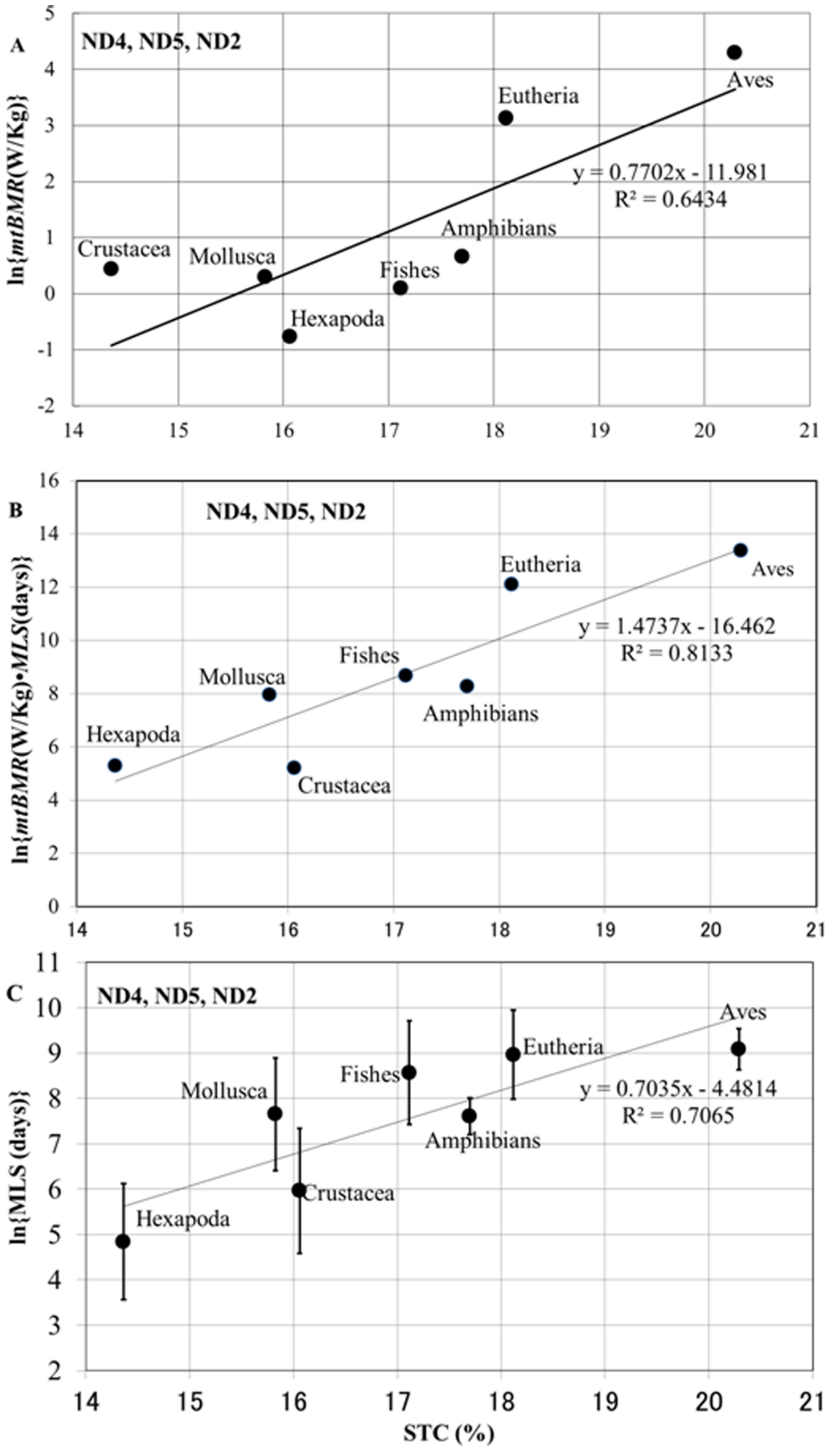
Relationships of *STC* with *mtBMR* (A), *mtBMR*·*MLS* (B), and *MLS* (C).

We examined correlations between *mtBMR* and MMP variables (*TC*, *STC*, and *HYD*) at the same mt function level, by increasing the *F* value from 1.0 (corresponding to *msBMR*) to infinity. The *mtBMR* values were estimated by extending *msBMR* in the respective animal groups (Materials and Methods). Since the data on metazoans with low *BMR* were very limited, we here applied the recent data reported by Makarieva et al. [1] and also the AnAge database for vertebrates (Table S2). We investigated the correlation between *STC* and *mtBMR* by changing the *F*-value included in this quantity 1 from 1.0 to infinity (Materials and Methods). Then we found that excluding the *M*-dependence of *mtBMR* with *F* = ∞ provides the strongest correlation (Figure S3). As a result, *mtBMR* correlated significantly with all MMP variables (*STC*, *TC*, *HYD*, and *CC*) in almost all of the protein sets, whereas *msBMR* weakly correlated with *STC* in only the 5-, 6-, and 7-protein sets. Here, *mtBMR* showed an especially strong correlation with *STC* in all protein sets (Table 2), since *STC* well describes vertebrates [16] and this analysis includes relatively many vertebrates. Figure 4A demonstrates a typical case of the 3-protein set, which shows a significant *STC*-*mtBMR* correlation with markedly high *mtBMR*–values in Aves and Eutheria. We here note that the *STC*-*msBMR* correlation was not strong with R^2^ = 0.28.

### E) Correlations of *STC* with *MLS*

The significant correlation between *STC* and *mtBMR* prompted us to examine the relationship between *STC* and *mtBMR*·*MLS*, because *mtBMR*·*MLS* corresponds to the total consumption energy per mitochondrion during the time (*MLS*) and may therefore be interpreted as a performance of the mt function. Here, *MLS* is redefined as the mean value of the individual MLSs in an animal group. By taking account of this time effect, the *STC*-*mtBMR*·*MLS* correlation (R^2^ = 0.81) became much stronger than the *STC*-*mtBMR* correlation (R^2^ = 0.64) (the 3-protein set in Table 2), and separated vertebrates from other animal groups (Figure 4B). Figure 4C demonstrates a strong *STC*-*MLS* positive correlation (R^2^ = 0.71) in the 3-protein set, and we found significant correlations between the MMP variables and *MLS* (Table 2). The *CC*-*MLS* correlation is likely to be related to oxidative damage to mitochondrial proteins or mtDNA [22]–[24], but was always weaker than the *STC*-*MLS* correlation in all protein sets (Table 2).

## Discussion

### Gradual increase in *HYD* for natural selection in many animal groups

A recent structural elucidation of ion channels in transmembrane proteins has provided evidence that these proteins undergo conformational changes during their function [13]. An easily understandable strategy of ecological and natural selection in metazoans is a reduction in their *BMR* by utilizing the multicellular effects of allometric scaling. Indeed, in many animal groups except for the deuterostomes, *HYD* and *TC* gradually increased and decreased, respectively, with decrease in *TSN* (Figure 3). Here, the decrease in *TC* correlated with that in *mtBMR* (Figure 4A), since less dynamic mobility and weaker stability in MMPs balance each other for the mt function. The expression of animal groups in terms of *HYD* and *TC*/*STC* may be consistent with their phylogenetic tree. Indeed, the order of animal groups along the *TSN*-*HYD* regression line in Figure 3 became globally compatible with the branching pattern projected on the evolutional pathway from Porifera toward Platyhelminthes in the molecular (rRNA)-based phylogeny reported by Adoutte et al. [21]. This compatibility was supported by the neighbor-joining tree [25] in terms of *TSN* and *TC* (Figure S4) and also by a multidimensional vector space method of tree building (Figure S5) [26], [27]. However, the 2 lowest values of *TSN* were occupied by Platyhelminthes (Acoelomata) and Nematoda (Pseudocoelomata). The life style of these two groups seems to be closely related to each other, since they live mostly in anaerobic environments. On the other hand, the molecular-based phylogeny coupled Platyhelminthes with Mollusca (Coelomata) as Lophotropods. Apart from this problem, the gradual increase in *HYD* of MMPs well explains the evolutionary pathway of ecological selection to strengthen their stability in many animal groups except for the deuterostomes.

### Increase in *STC* and decrease in *HYD* for adaptive evolution in Deuterostomes

The marked decrease in *HYD* and increase in *STC* of the deuterostome lineage are quite interesting (Figure 3), because they are likely to break the ordinary trade-off relationship rule between mobility and stability as being well understood in the evolutionary pathway of many other animal groups. These mutually reverse pathways of *HYD* and *STC* in the 2 large animal groups are far from regarding mtDNA as the neutral marker long held to be [28], cannot be explained by the nucleotide mutation pressure [29]–[32], and are therefore a strong evidence of adaptive evolution at the mt genome level. Another reverse process was previously observed in vertebrate marine animals such as cetaceans and alligators which returned from the land to water, because these animal groups underwent the evolutionary pathway of an increase in *HYD* and a decrease in *STC* toward Fishes in contrast to that of the decrease in *HYD* and increase in *STC* [16].

### Marked decrease in *HYD* and increase in *STC* heighten mt function in vertebrates

To pursue the biological meaning of the decrease in *HYD* and increase in *STC* in the deuterostome lineage, we introduced the quantity, *mtBMR*, being an energetic function at the same mt level as *HYD* and *STC*. Indeed, *mtBMR* was correlated negatively with *HYD* and positively with *STC* (Table 2). Figure 4A demonstrates a typical *STC*-*mtBMR* correlation (R^2^ = 0.64) in the 3-protein set (a linear combination of *STC* and *HYD* provided a stronger correlation (R^2^ = 0.77) with *mtBMR*). The marked decrease in *HYD* supports the appearance of a very large *mtBMR* in Aves and Eutheria, since a highly active mt-function can be attained by realizing MMPs with greater conformational freedom. However, on the other hand, higher MMP instability induced by this greater freedom heightens turnover rates of mitochondria, which requires a higher cost to reproduce a large number of them within cells. Furthermore, spatial constraints in metazoan tissues make it difficult for organisms to develop much higher power by simply accumulating more mitochondria, because mitochondria in metabolically active cells (such as those of the liver and brain) of, for example, humans make up 40 percent of the cytoplasm [17]. In this situation, the marked increase in *STC* in MMPs of Aves and Eutheria must be a critical condition to compensate or overcome their instability.

### The reason why Ser and Thr residues can enhance dynamic stability of MMPs

Interestingly, membrane proteins have an outstanding feature of being able to strengthen their dynamic stability by interhelical interactions between motifs involving moderately polar residues such as Ser and Thr [13]–[15]. Indeed, the decrease in *HYD* and increase in *STC* in Aves and Eutheria were markedly large within the membrane itself, as is well understood by comparing their differences between Aves/Eutherians and Platyhelminthes/Nematoda (Figure 2). It is therefore likely that the increase in *STC* corresponds to increased hydrogen bonding between helices, within and between subunits, as pointed out by Dawson et al. [15] and Hildebrand et al. [13]. Because Ser and Thr residues are small and only moderately polar, helical structures tend to be stabilized by cooperative networks of interhelical hydrogen bonds. In a previous paper [16], we showed that the short-range force of hydrogen bonds (1–2 Å) can be extended 2 to 3-fold (on average) by dynamic conformational changes in MMPs, because the relative distance of hydrogen bonding oscillates with the average amplitude R around R. Such a long-range potential amplifies the probability of interhelical interactions (in three-dimensional space) between cooperative networks of hydrogen bonds between motifs involving Thr or Ser residues. We envisage that such dynamic interactions could enable rapid resonance between metastable conformational states in MMPs, which have individual enzyme turnover rates of tens to hundreds of electrons per second [33]. In contrast, other types of hydrogen bonding, such as Cα-H---O hydrogen bonding between Gly and Ala residues and the helical backbone, produce more rigid structures [13].

### Proton-pumping machinery and dynamic conformational changes in MMPs

Three large subunits (ND2, ND4 and ND5) in complex I provided the strongest correlations between *TC* and *HYD* (Table 1), which were further correlated with *mtBMR* (Table 2). These subunits exhibit homology with sodium-proton antiporters and are known to be part of the proton pumping machinery of complex I [34]. Structural models of complex I suggest that electron transfers in the hydrophilic matrix arm are coupled to proton translocation in the membrane arm: a redox-dependent conformational change around the Q-site is transmitted to the 3 antiporter-like proton-pumping subunits (ND2, ND4 and ND5). In this way, proton translocation in complex I requires dynamic conformational changes across several subunits [4], [5], [8], [9]. Likewise, the 2 large subunits (CO1 and CO3) of complex IV, which form the second group of the strong *TC*-*HYD* correlations, may transfer protons across the membrane via conformational changes induced by electron transport [10], [11]. A similar relationship is also true for the proton-pumping subunits in complex III (CYTB), which is directly involved in proton-pumping via the Q-cycle. The precise mechanism of proton pumping via the Q cycle is uncertain while shuttling electrons and protons across the membrane implies a lower requirement for dynamic conformational changes in CYTB. We do indeed report a less tight correlation between *HYD* and *TC* in CYTB (Table 1). From the above-mentioned arguments, we speculate that the marked decrease in *HYD* and increase in *STC* (*TC*) in Aves and Eutheria arranged a fundamental condition to afford the powerful and robust proton-pumping machinery of complexes I and IV in these groups.

### The reason why *STC* is relevant to *mtBMR* and *MLS*

The mt energy power profoundly influenced the origin and evolution of the eukaryotic cell [35], [36], and also must be closely associated with *BMR* to sustain organismal life. We noticed that variations of *mtBMR* across the different animal groups may be shielded by the very strong *M*-dependence in the allometric scaling law (as demonstrated in Mammals and Aves of Figure S6). Therefore, by defining *mtBMR* so as to minimize its *M*-dependence, we obtained a significant correlation between *mtBMR* and *STC* in contrast to a weak correlation between *msBMR* and *STC* (Table 2). The markedly large values of *mtBMR* and *STC* in Aves and Mammals strongly suggest that high degrees of dynamic conformational changes and stabilization of MMPs are realized in these animal groups, so that this stabilization effect may lower the turnover rates of mitochondria and cells, and ultimately influence organismal lifespan as well as aerobic capacity. Indeed, we obtained a significant correlation between *STC* and *MLS*, despite the enormous variations [37] in lifestyles among the different animal groups. When we recall that 10 million billion mitochondria exist in an adult human [17] and that the resources for their activation are supplied from the host cells, the stability of MMPs affecting the turnover rate of mitochondria can be a fundamental factor to sustain human life

A few animal groups of Eutheria such as rodents and insectivores (with a very high *BMR*) do not show significant *STC*-*HYD* and *STC*-*MLS* correlations [16]. These animal groups are considered to have developed a life strategy to ensure survival by countering a short longevity with quite high reproduction rates. Such behaviors in rodents and insectivores sharply contrast to those of primates with a long longevity, because a very high amino-acid replacement rate in the simian lineage is accompanied by a marked increase in *TC* and decrease in *HYD* [38]. These observations teach us that the pattern of the mt adaptive evolution is not unique even among vertebrates.

### STC describes the vertebrate behavior better than TC

The potential penalties for introducing polar residues into hydrophobic membrane proteins may explain the fact that a strong inverse correlation between *HYD* and *TC* was observed across all metazoans in this study and indeed was stronger than the correlation between *HYD* and *STC*. In contrast, in our earlier studies on primates [38] and vertebrates [16], we observed strong inverse correlations between *HYD* and *STC*. The weaker correlation reported here could relate to 2 facts: the Ser residue is more polar than the Thr residue and hence is more difficult to incorporate into very hydrophobic proteins, and the hydrophobicity of MMPs is much greater in basal metazoans such as sponges and nematodes than in vertebrates. Thus it might be relatively easy to substitute Thr residues (compared with Ser) into the MMPs of basal metazoans. This interpretation is supported by the fact that substitutions of polar residues are the most common disease-causing mutations in membrane proteins, in part through altering bilayer partitioning, but also by altering function [39]. Presumably, the more hydrophobic the membrane protein, the more problems are caused by substitution of polar residues. Conversely, the less hydrophobic the protein, the less problematic is the insertion of more polar residues such as Ser. Accordingly, we note that Ser enrichment becomes more marked in the less hydrophobic MMPs of vertebrates.

### Overall comments about the results

Thus, overall, our findings are consistent with the hypothesis that cooperative networks of hydrogen bonds involving Thr and Ser residues stabilize dynamic conformational changes in MMPs, presumably increasing aerobic capacity, although we have not measured that directly. Direct measurements of the effect of increased *TC* or *STC* on MMP catalytic efficiency (Kcat), either *in vitro* or *in vivo*, are very difficult, as each substitution is likely to be highly dependent on the context. Cryptic epistasis is common in molecular evolution [40], and the requirement for multiple interactions with nuclear as well as mitochondrial genes [41], [42] only makes the problem more extreme in the case of respiratory proteins. Moreover, respiratory flux can be increased by adaptations throughout the entire supply network, including lung structure, hemoglobin kinetics, and capillary density [43], [44], as well as substrate channeling via respirasome assembly [45]. Given this complexity, the pervasive correlation between *TC* (S*TC*) and *HYD* in MMPs right across metazoans stands as strong evidence that selection for aerobic capacity at the level of mitochondrial-encoded subunits has indeed taken place. This view is consistent with a number of studies indicating regular selective sweeps on mitochondrial genes: mtDNA is far from the neutral marker it was long held to be [46].

### Remaining problems

It was difficult to detect the species-to-species coincidence between the sequence data and the observed data on *BMR* and/or *MLS*. Therefore, we used the average values of these data in respective animal groups without taking account of this coincidence. More available data in future will provide a clearer relationship between the MMP variables and *mtBMR*/*MLS* in more animal groups. We did not perform temperature adjustments of metabolic rates. One reason is that a common measurement temperature does not exist because endothermic groups do not live at body temperatures of 25°C as in many other animal groups. Another reason is that metabolic rate and temperature are not independent variables with each other, but may be rather correlated.

## Conclusion

The deuterostome lineage presented a quite unique evolutionary pathway of a marked decrease in *HYD* and increase in *STC*, in sharp contrast with the pathway of many other animal groups showing a gradual increase in *HYD* and decrease in *STC*, reflecting the natural selection to utilize the multicellular effect. These decreases in *HYD* and increases in *STC* were remarkable in the 5 large subunits (ND4, ND5, ND2, CO1 and CO3) in complexes I and IV, which require their dynamic conformational changes to exert a high degree of proton-pumping function. The low *HYD* values for these subunits are congruent with the large *mtBMR* values associated with their dynamic mobility. Furthermore, the marked increase in *STC* can strengthen dynamic stability of them via helix-helix interactions. As a result, this dynamic stability can lower the turnover rate of mitochondria and cells, and ultimately prolong the lifespan of organisms. In this way, vertebrates (especially Aves and Mammals) are considered to have equipped an excellent mechanism of action in MMPs to attain both very high metabolic rate and long longevity.

## Supporting Information

Figure S1 **Global relationship between** ***TC*** **and** ***HYD*** **in MMPs throughout metazoans.** Strong correlations with (R^2^>0.9) were obtained by analyzing all 13 proteins with S>0 (see Materials and Methods).
(TIF)

Figure S2 **The** ***TSN*** **–dependence of the animal groups in various protein sets.** This figure shows that the relative positions of the 13 animal groups are invariant in any protein sets of **1**) 3-protein set (ND4, ND5, ND2), **2**) 4-protein set (ND4, ND5, ND2, ND1), **3**) 5-protein set (ND4, ND5, ND2, CO1, CO3), **4**) 6-protein set (ND4, ND5, ND2, CO1, CO3, ND1), and **5**) 7-protein set (ND4, ND5, ND2, CO1, CO3, ND1, CYTB).
(TIF)

Figure S3 **The** ***F*** **-value dependence of correlation (R^2^) between** ***STC*** **and** ***mtBMR*** **.** The correlation (R^2^) between *STC* and *mtBMR* is estimated by changing the *F*-value included in this quantity from 1.0 to infinity (Materials and Methods). Here, *F* = ∞ excludes the *M*-dependence of *mtBMR* completely, and *mtBMR* depends on only the constant C in each animal group.
(TIF)

Figure S4 **Neighbor-joining tree in terms of** ***TC*** **and** ***TSN*** **.** We defined the pairwise distance between the i-th and j-th animal groups by *D*(i, j) =  {*TC*(i)-*TC*(j)}^**2^/σ_TC_^**2^ +{*TSN*(i)-*TSN*(j)}^**2^/σ_TSN_^**2^. *TC*(i) and *TSN*(i) denote the average values of *TC* and *TSN* in the i-th animal group, respectively, by using the 5- protein set (ND4, ND5, ND2, CO1, and CO3). σ_TC_ and σ_TSN_ denote the standard deviations of *TC* and *TSN*, respectively.
(TIF)

Figure S5 **Two dimensional display of a tree prepared by a multidimensional vector space method.** According to the multidimensional vector space (MVS) method for preparation of a phylogenetic tree [19], the molecular evolution of a tree branch is described as going into a new dimensional space, the direction of which is therefore perpendicular to that of the original pathway. For simplicity, let us consider a tree structure in 2-dimensional (X-Y) space, as illustrated in this figure. Here, the lineage **A** represents the main pathway from the tree root to species α, and the lineage B represents a branch pattern from the lineage **A** toward species β. When X and Y are variables independent from each other without any attractions (convergent evolution), the angle between the 2 lineages is 90^o^ (when they are closely correlated, the angle may be much deviated from 90^o^, as seen in the case of lines **B** and **C** of Figure 1 for the *HYD*-*TC*/*STC* strong correlation). The other species except for **A**, **B** and tree root must lie on the line **A** or **B** because they evolve into new dimensional spaces. If there are long-branch attractions, they fluctuate around these lines, as seen in this figure. We note that the lineage C may include many species but degenerates into one point in the X-Y plane and that the variables (to be used) must identify as many species as possible so as to explicitly describe a tree structure within these variables.
(TIF)

Figure S6 **The** ***M*** **-dependence of** ***BMR*** **in Aves and Mammals.**
(TIF)

Table S1 **The accession numbers of amino acid sequences of 13 animal groups.**
(XLSX)

Table S2 **A list of** ***msBMR*** **in the7 metazoan animal groups.** The 4 quantities of *msBMR*, scaling exponent α and constant C is defined in Materials and Methods.
(XLSX)
