# Supplementary figures and images for: Evolution of Mitochondrial Power in Vertebrate Metazoans
Source: PLoS One. 2014 Jun 9;9(6):e98188. doi: 10.1371/journal.pone.0098188 (PMC4049578; doi:10.1371/journal.pone.0098188)

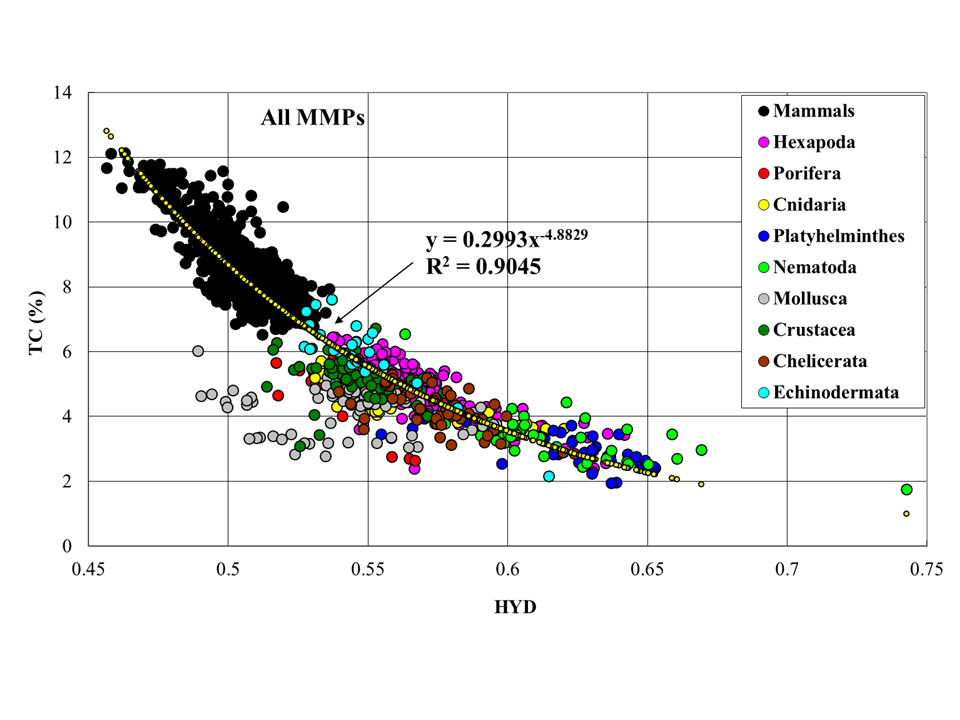

Supplement: Figure S1 — Global relationship between TC and HYD in MMPs throughout metazoans. Strong correlations with (R2>0.9) were obtained by analyzing all 13 proteins with S>0 (see Materials and Methods). (TIF) [file pone.0098188.s001.tif]

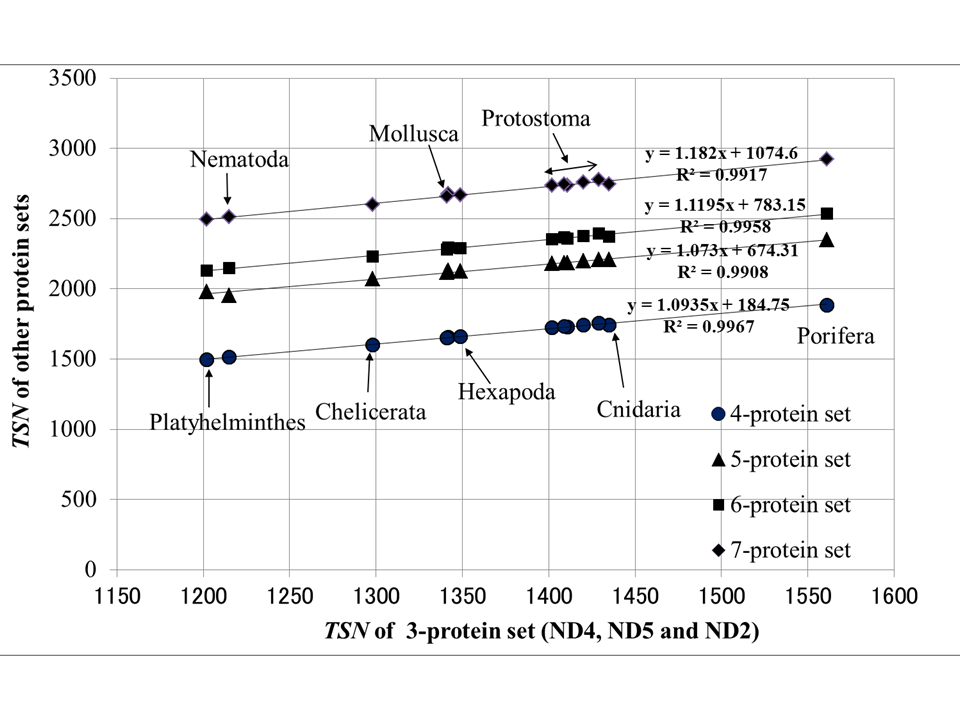

Supplement: Figure S2 — The TSN –dependence of the animal groups in various protein sets. This figure shows that the relative positions of the 13 animal groups are invariant in any protein sets of 1) 3-protein set (ND4, ND5, ND2), 2) 4-protein set (ND4, ND5, ND2, ND1), 3) 5-protein set (ND4, ND5, ND2, CO1, CO3), 4) 6-protein set (ND4, ND5, ND2, CO1, CO3, ND1), and 5) 7-protein set (ND4, ND5, ND2, CO1, CO3, ND1, CYTB). (TIF) [file pone.0098188.s002.tif]

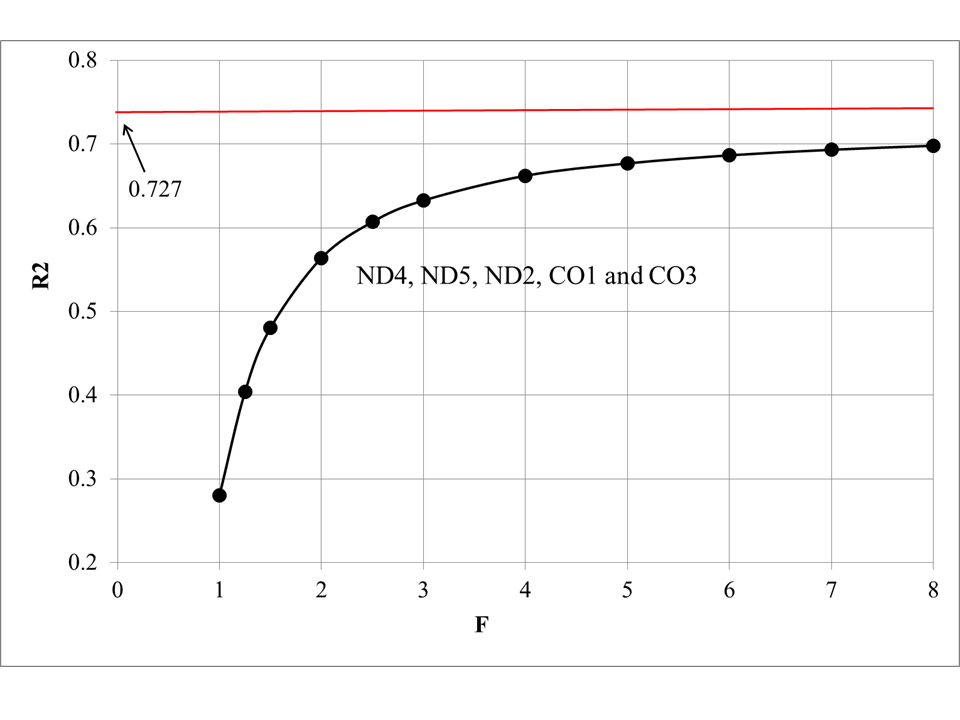

Supplement: Figure S3 — The F -value dependence of correlation (R2) between STC and mtBMR . The correlation (R2) between STC and mtBMR is estimated by changing the F-value included in this quantity from 1.0 to infinity (Materials and Methods). Here, F = ∞ excludes the M-dependence of mtBMR completely, and mtBMR depends on only the constant C in each animal group. (TIF) [file pone.0098188.s003.tif]

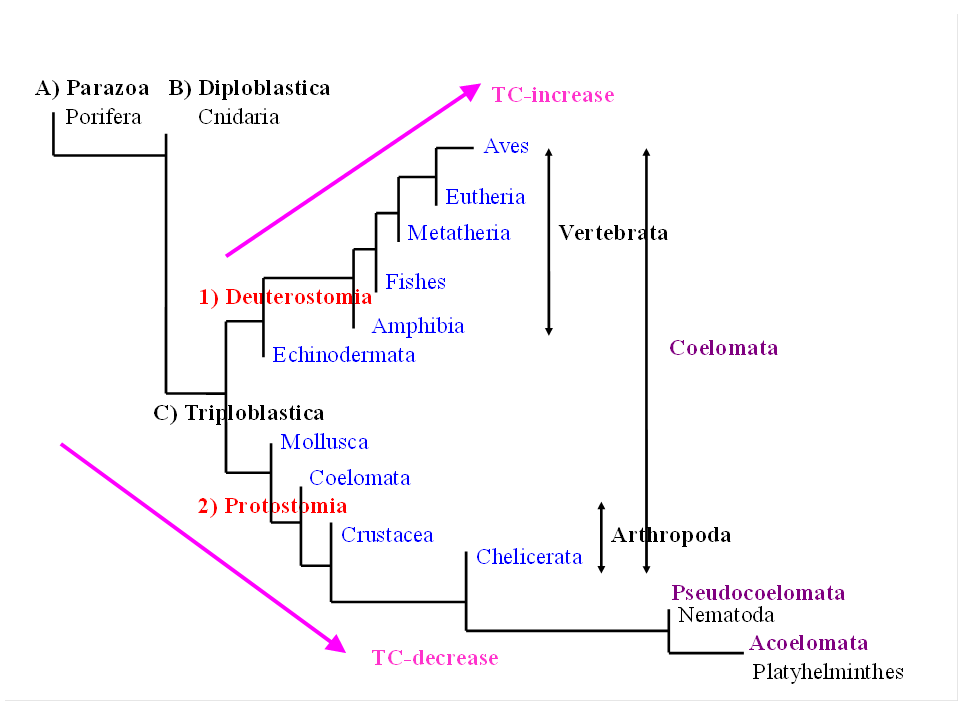

Supplement: Figure S4 — Neighbor-joining tree in terms of TC and TSN . We defined the pairwise distance between the i-th and j-th animal groups by D(i, j) = {TC(i)-TC(j)}**2/σTC **2 +{TSN(i)-TSN(j)}**2/σTSN **2. TC(i) and TSN(i) denote the average values of TC and TSN in the i-th animal group, respectively, by using the 5- protein set (ND4, ND5, ND2, CO1, and CO3). σTC and σTSN denote the standard deviations of TC and TSN, respectively. (TIF) [file pone.0098188.s004.tif]

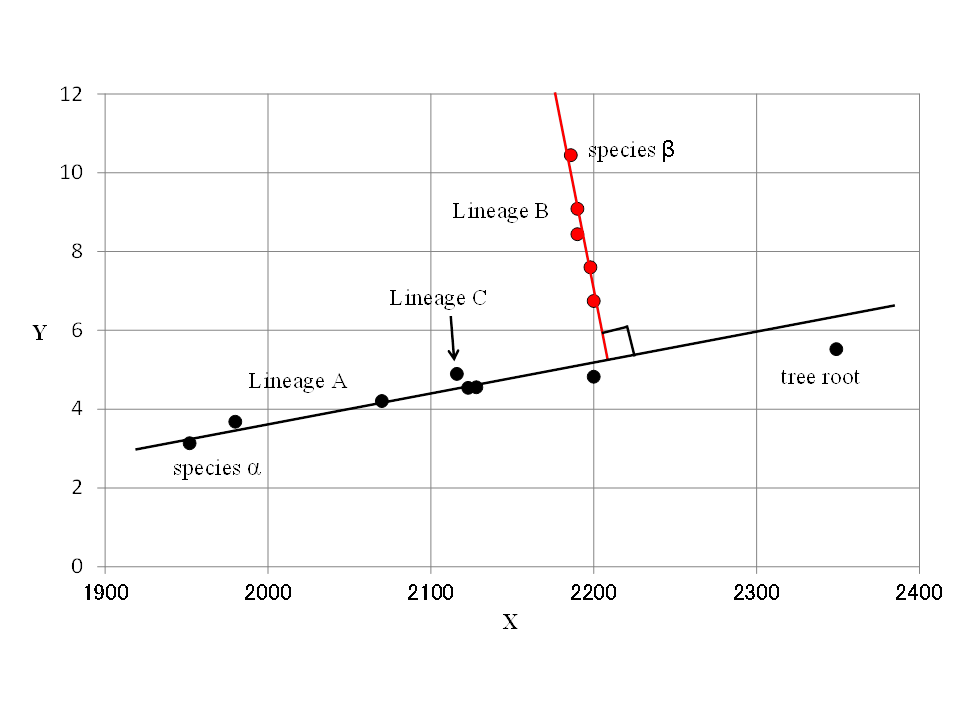

Supplement: Figure S5 — Two dimensional display of a tree prepared by a multidimensional vector space method. According to the multidimensional vector space (MVS) method for preparation of a phylogenetic tree [19], the molecular evolution of a tree branch is described as going into a new dimensional space, the direction of which is therefore perpendicular to that of the original pathway. For simplicity, let us consider a tree structure in 2-dimensional (X-Y) space, as illustrated in this figure. Here, the lineage A represents the main pathway from the tree root to species α, and the lineage B represents a branch pattern from the lineage A toward species β. When X and Y are variables independent from each other without any attractions (convergent evolution), the angle between the 2 lineages is 90o (when they are closely correlated, the angle may be much deviated from 90o, as seen in the case of lines B and C of Figure 1 for the HYD-TC/STC strong correlation). The other species except for A, B and tree root must lie on the line A or B because they evolve into new dimensional spaces. If there are long-branch attractions, they fluctuate around these lines, as seen in this figure. We note that the lineage C may include many species but degenerates into one point in the X-Y plane and that the variables (to be used) must identify as many species as possible so as to explicitly describe a tree structure within these variables. (TIF) [file pone.0098188.s005.tif]

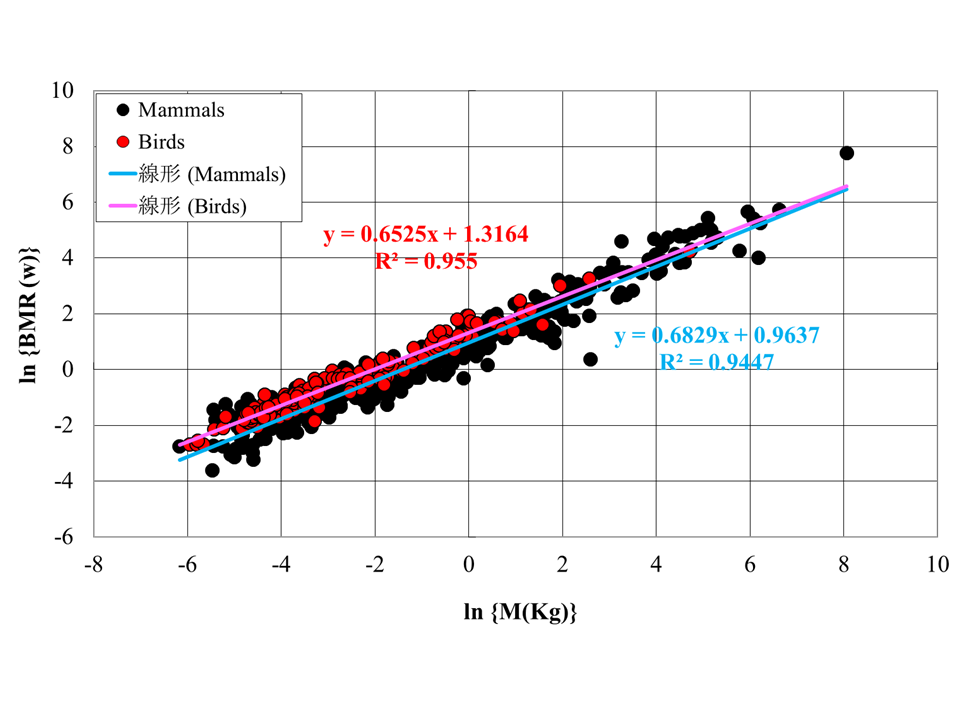

Supplement: Figure S6 — The M -dependence of BMR in Aves and Mammals. (TIF) [file pone.0098188.s006.tif]
